# DNA methylation-based measures of accelerated biological ageing and the risk of dementia in the oldest-old: a study of the Lothian Birth Cohort 1921

**DOI:** 10.1186/s12888-020-2469-9

**Published:** 2020-02-28

**Authors:** Ruth A. Sibbett, Drew M. Altschul, Riccardo E. Marioni, Ian J. Deary, John M. Starr, Tom C. Russ

**Affiliations:** 1 Alzheimer Scotland Dementia Research Centre, University of Edinburgh, 7 George Square, Edinburgh, EH8 9JZ UK; 2 Centre for Cognitive Ageing and Cognitive Epidemiology, University of Edinburgh, Edinburgh, UK; 3 Department of Psychology, University of Edinburgh, Edinburgh, UK; 4 Centre for Genomic and Experimental Medicine, Institute of Genetics and Molecular Medicine, University of Edinburgh, Edinburgh, UK; 5 Edinburgh Dementia Prevention Research Group, University of Edinburgh, Edinburgh, UK

**Keywords:** Dementia, DNA methylation, Accelerated ageing, Epigenetic age

## Abstract

**Background:**

Previous studies have demonstrated an association between DNA methylation-based measures of accelerated ageing and age-related health outcomes and mortality. As a disease closely associated with advancing age, we hypothesized that DNA methylation-based measures of accelerated ageing might be associated with risk for dementia. This study therefore aimed to examine the association between four recognised measures of age acceleration and subsequent dementia.

**Methods:**

Study subjects (*n* = 488) were members of the Lothian Birth Cohort 1921. Dementia case ascertainment used data from death certificates, electronic hospital records, and clinical reviews. Venous blood samples were taken at baseline, at age 79 years. DNA methylation and measures of epigenetic age were calculated in accordance with Horvath’s epigenetic clock tutorial, using the online calculator (https://dnamage.genetics.ucla.edu/). From these values, four measures of accelerated ageing were calculated: extrinsic epigenetic age acceleration (EEAA), intrinsic epigenetic age acceleration (IEAA), AgeAccelPheno and AgeAccelGrim. Competing risk regression models – with death as a competing risk – were performed to examine the association between each measure of accelerated ageing and incident dementia. *APOE* ɛ4 status, sex, age, smoking status, history of cardiovascular disease, cerebrovascular disease, hypertension, and diabetes were included as covariates.

**Results:**

None of the multivariate models revealed a positive association between increased epigenetic age acceleration and dementia risk. Across all included models, never-smoking increased risk for dementia (HR 1.69 [1.06, 2.71], *p* = 0.03), and having no *APOE* ɛ4 alleles reduced risk for dementia (HR 0.44 [0.29, 0.67], *p* < 0.001).

**Conclusions:**

The present study did not demonstrate any consistent association between DNA methylation-based measures of accelerated ageing and dementia in subjects aged over 79 years. Further, larger studies – including separate analyses of dementia subtypes – are required to further investigate the potential association between DNA methylation-based measures of accelerated ageing and dementia.

## Background

As the global population ages, diseases closely associated with advancing age are projected to increase in number as a result. Dementia is one such disease, the most common cause for which is Alzheimer’s disease (AD). As the number of dementia cases increases, so will the economic and social care requirements [1]. Managing the impact of dementia will therefore pose a significant public health challenge. Gaining a comprehensive understanding of the risk factors for dementia is a vital step in addressing this challenge.

It is recognised that both genetic and environmental factors contribute to the development of dementia. There is, however, considerable variability in the risk that one will develop the disease. It is thought therefore, that the aetiology is likely to be a complex interaction between genetic and environmental factors. Epigenetics could be considered to be a bridge between genes and the environment, with exposure to environmental factors giving rise to alterations in gene expression, via epigenetic mechanisms [2]. Unsurprisingly, the study of epigenetics is an area of considerable research interest and it may prove to be important in understanding dementia risk.

Older age is widely recognised to be the most significant risk factor for dementia. However, it is clear that some individuals age more successfully than others, in that, for some, advancing age has less effect on physical robustness, health (and disease) status and cognitive function [3, 4]. The explanation for individual differences in the effect of ageing is also likely to be multifactorial, with genetic, lifestyle and health factors all playing a role [3]. It has been suggested that each individual has a biological or physiological age that may differ from chronological age and is the result of such factors. Furthermore, studies have shown that each individual may have a series of biological ages, depending on the biomarker used to estimate age, suggesting that the biological ageing process may not just vary between individuals, but also within each individual [5].

Patterns of a specific epigenetic modification within the DNA sequence – DNA methylation – have been used in previous studies to calculate estimates of biological age. DNA methylation is one of the most frequently-studied epigenetic marks and occurs with the addition of a methyl group to the DNA molecule, typically at a cytosine nucleotide that precedes a guanine nucleotide – CpG sites [2]. Such estimates of age are typically referred to as the ‘epigenetic age’ or ‘DNAm (DNA methylation) age’ and are suggested to reflect both an individual’s biological age and their susceptibility to age-related health outcomes [6]. DNA methylation-based estimates of age have been shown to be consistent across biological sample types, including blood and various tissues [6]. Whereas epigenetic age has been shown to correlate highly with chronological age, significant discrepancies between the two are noted at the individual level [6]. Studies comparing chronological age with epigenetic age found that there was an increased risk of all-cause mortality for those exhibiting accelerated ageing – i.e. those who had higher epigenetic age than chronological age – after adjusting for related genetic, health and lifestyle factors [6, 7]. Furthermore, the offspring of persons surviving to 105–109 years of age have been shown to have a lower epigenetic age than age-matched controls [8]. A number of suggested risk factors for dementia have also been shown to be associated with greater age acceleration; poorer physical fitness, lower cognitive ability, lower socioeconomic status, greater body mass index, higher total cholesterol to high-density lipoprotein cholesterol ratios, hypertension and smoking (greater pack-years) have all been shown to be associated with greater age acceleration (calculated using DNA methylation-based measures) [9, 10]. Based on these previous findings, we hypothesized that measures of accelerated biological ageing based on DNA methylation would be a valuable predictor of dementia risk.

This study would consider four recognised DNA methylation-based measures of accelerated ageing: the two first-generation measures of age acceleration – intrinsic epigenetic age acceleration (IEAA) and extrinsic epigenetic age acceleration (EEAA) – and the two novel estimates of age acceleration – AgeAccelPheno (based on PhenoAge) and AgeAccelGrim (based on GrimAge). The rationale for including the older measures was that these have been the most consistently reported in the literature and there is therefore more evidentiary basis that these measures were valid and appropriate for inclusion in our study. The novel measures were included on the basis that these have been shown to be more accurate predictors of mortality, time-to-death and other morbidities than the earlier measures [11, 12].

The earlier measures – IEAA and EEAA – were based on methods for estimating epigenetic age described by Horvath [13] and Hannum et al. [14], respectively, in 2013 [6]. Horvath’s epigenetic age estimate is based on DNA methylation at 353 CpGs, while Hannum’s epigenetic age estimate is based on DNA methylation at 71 CpGs. Both age acceleration measures compare epigenetic age estimates with chronological age in order to define age acceleration. The age acceleration measures also differ however, in that IEAA is independent of changes in blood cell composition, whereas EEAA incorporates age-related changes in blood cell composition [6]. Whereas the epigenetic age estimates produced using these measures have demonstrated statistically significant associations with age-related conditions, the effect sizes seen have been relatively small [11].

Levine et al. proposed that a new, more successful DNA methylation-based measure of epigenetic age may be developed by using “phenotypic age” as a reference, rather than chronological age [11]. So-called “phenotypic aging measures” are based on clinical biomarkers (albumin, creatinine, serum glucose, C-reactive protein, lymphocyte percent, mean cell volume, red cell distribution width, alkaline phosphatase, white blood cell count) and age, and had previously been shown to be associated with differences in risk for mortality, physical and cognitive function, facial ageing and life expectancy [11]. In 2018, Levine et al. published the novel measure for epigenetic age, produced by regressing a phenotypic measure of mortality risk on CpGs: DNAm PhenoAge [11].

More recently, Lu et al. published another novel measure of epigenetic age, termed DNAm GrimAge [12]. In a two-step process, the authors began by identifying DNA methylation-based biomarkers of mortality and morbidity including several plasma proteins and smoking pack-years; time-to death was then regressed onto these biomarkers, producing a single composite biomarker of lifespan: DNAm GrimAge [12]. By adjusting the measure for chronological age, the authors produced a measure of age acceleration: AgeAccelGrim [12]. Each of the four measures are in units of year.

Given the differences in how each measure arrives at a calculation of epigenetic age acceleration, and the differences between measures in the accuracy of prediction for other outcomes shown in previous studies, one would not necessarily expect our results to be consistent between age acceleration measures. Because the novel methods have been shown to be more accurate predictors of morbidity and mortality in previous studies, we might expect that these measures would be more accurate in predicting incident dementia.

In summary, we explore the associations between four DNA methylation-based measures of accelerated ageing and *n* = 109 cases of incident dementia from a cohort of *n* = 488 individuals, who were healthy when recruited at age 79 years, and followed-up for approximately 16 years. Given the known association between accelerated ageing and mortality, we recognised the potential for death to affect our findings. Death is therefore considered as a competing risk in our analyses.

## Methods

### Participants

Participants were members of the Lothian Birth Cohort 1921 (LBC1921), recruited from 1999, with baseline testing at mean age 79 years. The cohort has been described in detail within the literature and an overview will be provided here [15, 16]. All participants were born in 1921, and most had taken part in a general intelligence test at age 11 years – the Scottish Mental Survey 1932 (SMS1932) [17, 18]. The survey was completed within Scottish schools and used a validated test of intelligence. SMS1932 participants were recruited for follow-up in later life, with the aim of investigating the possible determinants of non-pathological cognitive ageing [19]. Five-hundred and fifty relatively healthy and independently living participants, residing mostly in and around the Lothian area of Scotland, enrolled in the study and attended baseline testing. Surviving participants who remained in the study were re-tested at four subsequent test waves; at approximately 83, 87, 90 and 92 years of age [16]. Test waves used questionnaires and in-person testing and collected medical, physiological, genetic, cognitive, psychological and socio-demographic data. Information regarding participants who had died was provided at regular intervals by the General Registrar’s Office, Scotland.

Only those participants scoring 24 or higher on the Folstein Mini Mental State Examination (MMSE) [20] at baseline (*n* = 539) were included in the present study. Similarly, those reporting a history of dementia at baseline (*n* = 2) were not included. These exclusions were made in order to minimise the possibility that we were including prevalent cases of dementia in our analyses. Without such exclusions there is the possibility that we could falsely identify an association between epigenetic age acceleration and risk for incident dementia – when we were in fact identifying an association between epigenetic age acceleration and existing dementia. Ethical approval for the study was provided by the Lothian Research Ethics Committee (test waves 1–3) and the Scotland A Research Ethics Committee (test waves 4–5). From wave 4 onwards, participants were asked to provide consent for data linkage and access to health records.

### Measures of DNA methylation

Blood samples extracted at wave 1 (mean age 79) were used in the present study. DNA was extracted from whole blood samples at MRC Technology, Western General Hospital, Edinburgh, UK. Methylation typing was performed at the Welcome Trust Clinical Research Facility, Western General Hospital, Edinburgh. DNA samples were bisulphite converted and hybridised to the 12 sample Illumina HumanMethylation450BeadChips using the Infinium Methylation protocol and Tecan robotics.

Extensive quality control was conducted, as reported in Zhang et al., [21] to leave a dataset consisting 470,278 CpG sites from 436 LBC1921 participant observations. Briefly, one sample from each duplicate pair (same sample from the same wave) was removed, along with one sample from each replicate pair (same sample, different analysis set). Samples and CpG sites with low call rates (95% of CpGs and samples with *P* < 0.01) were excluded, as were XY probes.

Following this initial screening process, the raw IDAT files for these 436 individuals underwent a separate quality control analysis. This was conducted in accordance with the recommended analysis procedure in Hovarth’s epigenetic clock tutorial (https://dnamage.genetics.ucla.edu/), to help reduce missing CpG values. Raw DNAm IDAT files were read into R, using minfi, and were normalised using the noob (normal-exponential convolution using out-of-band probes) method, implemented by the preprocessNoob() function. This method estimates background noise from out-of-band probes and removes it for each individual sample; and performs dye-bias normalisation whereby a subset of control probes estimate the dye bias. The getBeta() function of minfi was used to obtain noob-normalised methylation beta values.

### Measures of epigenetic age

The online calculator developed by Hovarth (https://dnamage.genetics.ucla.edu/) was used to determine measures of epigenetic age (Intrinsic Epigenetic Age, Extrinsic Epigenetic Age, DNAm GrimAge, and DNAm PhenoAge) from the beta values described above. The age calculator performed a further normalisation process on the LBC1921 methylation data entered into the algorithm. Age acceleration measures were obtained for PhenoAge and GrimAge by extracting residuals from the model of epigenetic age on chronological age.

Intrinsic epigenetic accelerated aging (IEAA), and extrinsic epigenetic accelerated ageing (EEAA) have been described in detail within the literature by Chen et al. [6] IEAA is defined as the residual that resulted from a multivariate regression of epigenetic age – calculated using the Hovarth epigenetic age measure – on chronological age and measures of blood cell counts [6]. EEAA was based on the epigenetic age calculated using the measure described by Hannum et al., with a weighted average of Hannum’s age estimate being produced in order to increase the contribution of certain blood cell types (known to change with age) on the age estimation [6]. The resulting age estimate was regressed on chronological age in a univariate model, with EEAA representing the resulting residual variation [6].

### Additional variables

Covariates included in the main statistical models were as follows: age, sex, *APOE* ɛ4 carrier status, ever-smoking status, history of hypertension, history of diabetes and history of either cardiovascular or cerebrovascular disease. Genomic DNA was isolated from participants’ venous blood in order to determine *APOE* ɛ4 status. Participants were classified as carriers if they possessed one or more *APOE* ɛ4 alleles. Date of birth, sex, smoking history and history of hypertension, diabetes, cerebrovascular, and cardiovascular disease were self-reported by participants at the first wave of testing. Age was calculated as the number of days between date of birth and date of attendance at wave 1 testing. Additional analyses were performed to further investigate the component parts of AgeAccelGrim; additional covariates therefore included the DNAm-based surrogates for seven proteins and smoking pack years (beta-2 microglobulin (DNAm B2M), cystatin-C (DNAm Cystatin C), growth differentiation factor 15 (DNAm GDF-15), plasma activator-inhibitor 1 (DNAm PAI-1), Leptin (DNAm Leptin), adrenomedullin (DNAm ADM), and tissue inhibitor metalloproteinase 1 (DNAm TIMP-1) and DNAm PACKYRS.

It was important to determine the association between chronological age and dementia, before exploring whether DNA methylation-based measures of accelerated aging could be of greater predictive value in assessing dementia risk, hence the inclusion of chronological age in the analyses. Given the narrow-age nature of our cohort, there is little variance in age and we would not expect to observe a statistically significant association between age and dementia; the chronological age variable would not therefore be included in subsequent statistical models if this assumption was confirmed. *APOE* ɛ4 carrier status was included because of the known association with dementia, particularly as this association had been replicated in earlier studies of this cohort [22]. Smoking status was introduced given the recognised effect that smoking has on DNA methylation [23], and the potential for this to affect the findings. Furthermore, whereas smoking had not been found to be associated with dementia in previous studies of this cohort, it has been reported to be an important risk factor within the literature. A history of hypertension, diabetes and cardiovascular or cerebrovascular disease were included given the potential association with earlier death and dementia. Furthermore, such health outcomes increase with advancing age and so greater epigenetic age could be associated with susceptibility to these conditions [24, 25]. An interaction term for sex and measure of accelerated ageing was included as sex is strongly linked to both AgeAccelPheno and AgeAccelGrim. Whereas other factors – such as age 11 IQ – could be proposed to be associated with dementia [26], such variables have not been shown to be important with regard to dementia risk in this cohort and were not therefore included [22, 27]. Our hypothesis-driven approach, based on previous findings aimed to minimise the inclusion of variables that would not be relevant in this sample, and reduce the possibility of multiple hypotheses testing.

### Dementia ascertainment

Dementia case ascertainment in LBC1921 has been described previously in detail [22]. Briefly, cases were ascertained retrospectively, up to age 95 years, based on evidence collected from death certificates, medical records, and a small number of clinical assessments [22]. Death certificates available by the end of June 2016 were examined for any recording of either dementia or cognitive decline, in any position. For each participant who consented to data linkage and access to records, local electronic hospital records were reviewed and any evidence for dementia or cognitive decline was collected. Prior to 2014, psychiatric records were held on a separate electronic system and diagnoses were supplied to the study in the form of ICD-9 and ICD-10 codes for those who had been in contact with psychiatric services. ICD-9 and 10 codes that were relevant to the dementia ascertainment process are shown in Additional file 1: Figure S1. Latterly, psychiatric records were merged with the general hospital system and accessed as previously described. Data for each consenting participants were accessed using their Community Health Index (CHI) number, a unique identifier specific to each NHS patient in Scotland and recorded at each contact. The last date for data collection from medical records was the 16th of May 2016. Additional evidence was available for a small proportion of participants (*n* = 26) who underwent clinical review by one of the authors (TCR, JMS), either in the NHS or research setting. Any participant who reported a new diagnosis of dementia at routine LBC1921 follow-up, or any participant for whom a concern was raised regarding cognitive decline, was referred for such clinical assessment. Data from such reviews were collected up to 15th December 2016, when all of the evidence gathered was reviewed and discussed at a final dementia diagnosis consensus meeting (RAS, TCR, JMS). The meeting agreed upon the presence of a diagnosis and the subtype, using a previously described list of criteria for ‘probable’ or ‘possible’ dementia diagnosis [22]. Any disagreement was resolved through discussion. To minimise the potential for introducing classification error to the results, possible dementia cases were excluded from the analyses.

### Time-to-event variables

The events included in this study are dementia and death, determined as described above. The number of days between the date of attendance at wave 1 testing and date of death gave the ‘time to death’. For those who did not die, the censoring time was taken as the number of days between wave 1 testing and a date beyond that last date of data collection for any participant (6500 days after baseline testing). The number of days between the date of attendance at wave 1 testing and the first date that a dementia diagnosis was noted in any of the available sources gave the ‘time to dementia’. Where dementia was recorded on a death certificate and no duration was given, and dementia was not recorded in another source, the diagnosis was presumed to predate death by six months. Where the duration was not given, but a diagnosis was recorded in another source, the earliest such date was used to determine the date of onset. If sources recorded both cognitive impairment and dementia, the date of dementia onset was taken as the earliest recording of a dementia diagnosis. If dementia diagnosis was determined based on evidence that did not include a formal diagnosis of dementia, the earliest mention of cognitive impairment was used to date onset (as long as the same record did not specifically note the absence of a dementia syndrome). For participants who remained dementia-free, the ‘time to dementia’ variable was taken as either the time to date of death or to a date beyond that last date of data collection for any surviving participant (6500 days after baseline testing).

### Statistical analysis

The first step in analysing the data was to demonstrate any statistically significant differences (*p* < 0.05) between the group who developed dementia and the group who did not. Univariate analysis – using either the Pearson chi-square or t-test (*IBM SPSS, Version 21*) – was completed for each variable that would be included in the main analyses. The same software was used to calculate the level of correlation between the measures of epigenetic age acceleration. *R* statistical software, (package ‘cmprsk’ in *R version 3.5.1*) was used to perform all subsequent steps in the analyses.

The main analyses were completed using competing risk regression (CRR) models, in which death was considered a competing risk for dementia. Having been considered in a number of previous studies, the association between age acceleration measures and death would not be a primary focus of this study; incident dementia after age 79 years was the primary outcome to be reported. Death and dementia compete for risk in that they non-independently occur. This changes the risk function that a given variable may have with an outcome. For example, older individuals are likely to both die and get dementia. Two individuals might both die at the same time, before being diagnosed with dementia. One of these individuals would have developed dementia in a few months had they lived, the other would not have developed dementia for several years. In a more standard logistic or Cox model predicting only dementia diagnosis, both individuals would be censored out of the analysis at the time of death, and the information on the competing risk of death is ignored. Competing risk regression models take into account the information from a competing risk and reweights the primary outcome risk in light of competing outcomes. The first CRR regression model (CRR 1) explored the association between age and dementia, with chronological age (at baseline testing) being the only variable included. Chronological age was excluded from subsequent models as it did not prove to be statistically significant in this first model. The second model (CRR 2) examined measures of accelerated aging as a marker of biological ageing; the covariates included in the model were the given DNA methylation measure of accelerated ageing, sex, *APOE* ɛ4 carrier status, and a DNA methylation age acceleration and sex interaction term. The interaction term was to be excluded from subsequent models if it did not reach statistical significance. The third model (CRR 3) included these same variables, with the addition of ever-smoking status. The final model (CRR 4) included three additional health outcome variables – history of hypertension, history of diabetes, and history of cardiovascular or cerebrovascular disease. Accelerated ageing, sex and *APOE* ɛ4 are also included in this model. Smoking status is included if it was statistically significant in model 3.

The main findings of the CRR analyses were supported with cumulative incidence plots for each competing event (dementia and death); these illustrate the time-varying risk of dementia, between covariate levels. The Aalen-Johansen estimator was used to calculate the unbiased estimate of cumulative incidence. In addition to the CRR models, logistic regression models would be completed to establish the associations between variables and dementia, when death outcomes are not considered. The results would be made available within the supplementary materials.

## Results

### Cohort demographics

**Fig. 1:**
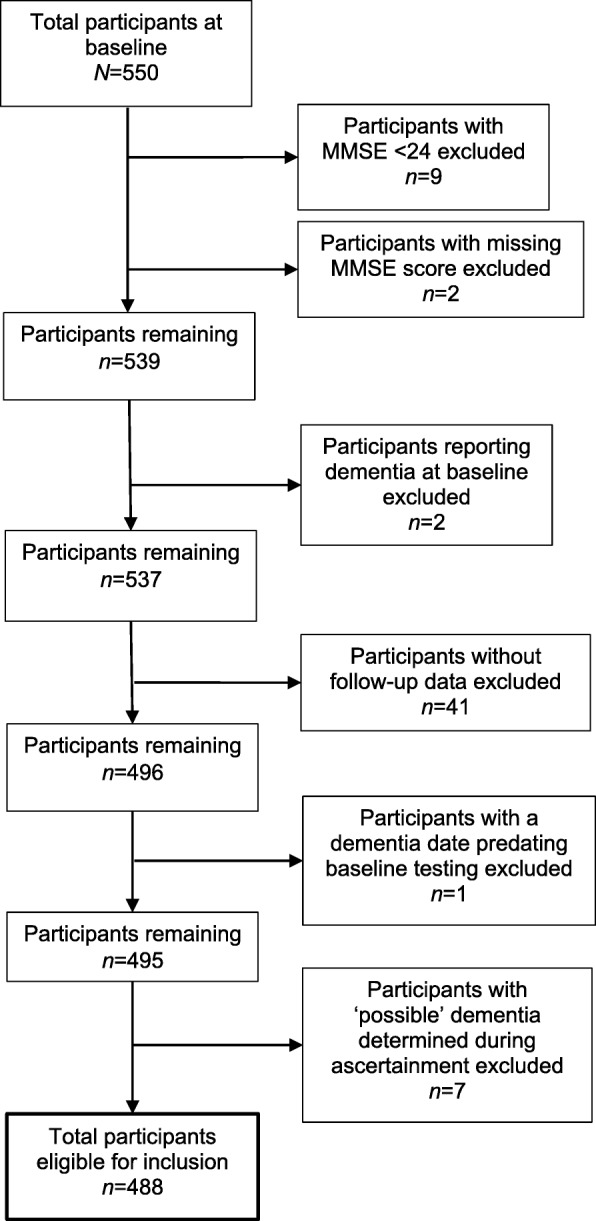
Flowchart for participant exclusion process

The complete LBC1921 cohort included *N* = 550 participants who were recruited and attended baseline testing at age 79 years. The participants eligible for these analyses did not include those who had an MMSE score of less than 24 at baseline (*n* = 9), those without a valid MMSE score at baseline (*n* = 2), those who reported a history of dementia at baseline (*n* = 2), and those with no follow-up data available for the purpose of dementia ascertainment (*n* = 41). For one participant, the calculated time to dementia suggested that dementia predated attendance at wave 1 testing and they were also excluded from the study sample. Of those who were eligible for inclusion in this study (*n* = 495), a consensus diagnosis of probable dementia was agreed for *n* = 109 and a consensus diagnosis of possible dementia was agreed for *n* = 7. Those with possible dementia were excluded from the analyses, resulting in a final study sample of *n* = 488 participants. Exclusions were made on a step-wise basis as shown in Fig. 1, with a total of *n* = 62 participants excluded from these analyses. Over half of the included participants were female (57.4%, *n* = 280) and over three-quarters were known to be deceased by the 30th of June 2016 (85.9%, *n* = 419). Of those who were deceased, 79.0% (*n* = 331) had died without a diagnosis of dementia. Descriptive statistics for those included and excluded are shown in Table 1, alongside group comparison statistics for those with and without dementia.

**Table 1:** Study sample demographics and univariate analyses

	Eligible Participants (*n* = 488)		Group Comparison *p* value (chi-square or t-test)	Excluded Participants (*n* = 62)
	Dementia (*n* = 109)	No Dementia (*n* = 379)		
Age	*n* = 109	*n* = 379		*n* = 62
-mean age in years (SD)	79.04 (0.55)	79.08 (0.59)	0.540	79.09 (0.53)
Sex	*n* = 109	*n* = 379		*n* = 62
% female	62.4%	55.9%	0.230	58.1%
Living or deceased	*n* = 109	*n* = 379		*n* = 62
% deceased	80.7%	87.3%	0.081	29.0%
MMSE score at baseline	*n* = 109	*n* = 379		*n* = 60
mean score (SD)	28.10 (1.64)	28.33 (1.46)	0.156	27.27 (2.67)
*APOE* ɛ4 carrier status	*n* = 109	*n* = 373		*n* = 61
% carrier *APOE* ɛ4	41.3%	22.5%	< 0.001	27.9%
Age 11 IQ (standardised)	*n* = 101	*n* = 339		*n* = 53
mean score (SD)	100.19 (16.18)	100.22 (14.53)	0.982	98.21 (15.63)
Smoking status	*n* = 108	*n* = 379		*n* = 62
% ever smoker	42.6%	61.7%	< 0.001	50.0%
Lifetime smoking packs^*^	*n =* 108	*n =* 376		*n = 58*
mean total packs (SD)	4359.83 (8016.04)	6616.27 (8740.85)	0.016	*3880.78 (6609.45)*
History of hypertension	*n* = 108	*n* = 375		*n* = 61
% positive history	35.2%	41.9%	0.212	41.0%
History of diabetes	*n* = 109	*n* = 379		*n* = 62
% positive history	4.6%	5.8%	0.624	1.6%
History of cardiovascular or cerebrovascular disease	*n* = 104	*n* = 373		*n* = 59
% positive history	28.9%	28.2%	0.889	22%
EEAA	*n* = 88	*n* = 295		*n* = 53
mean (SD)	0.37 (7.27)	2.35 (8.41)	0.047	−0.27 (6.68)
IEAA	*n* = 88	*n* = 295		*n* = 53
mean (SD)	−0.64 (5.60)	0.80 (6.84)	0.074	0.59 (5.13)
AgeAccelGrim	*n* = 88	*n* = 295		*n* = 53
mean (SD)	−1.30 (4.14)	0.66 (4.64)	< 0.001	−0.39 (4.91)
AgeAccelPheno	*n* = 88	*n* = 295		*n* = 53
mean (SD)	0.21 (6.56)	1.75 (7.63)	0.087	0.53 (6.55)

Note.^*^Lifetime smoking packs calculated by number of packs (20 cigarettes) smoked per year multiplied by the number of years smoking

### Dementia group comparison

Univariate analyses demonstrated little difference between those eligible participants who developed dementia and those who did not. Positive ever-smoking status (*p* < 0.001), greater smoking pack years (*p* = 0.016), increased DNAm GrimAge age acceleration (AgeAccelGrim) (*p* < 0.001) and increased extrinsic epigenetic accelerated ageing (EEAA) (*p* = 0.047) reduced the risk for dementia, while positive *APOE* ɛ4 (*p* < 0.001) carrier status increased the risk for dementia.

### Time-to-event variables

The mean time to dementia and the mean time to death for the eligible study sample were 3371.0 (SD: 1724.7) days and 3618.9 (SD: 1829.3) days, respectively. The mean time to death for deceased participants (*n* = 419) was 3144.5 days (SD: 1517.5). For the participants who survived, the ‘time to death’ variable value was taken as the number of days between baseline testing and a date beyond the last date of data collection for any participant; 6500 days. The mean time to dementia for those who developed dementia (*n* = 109) was 3535.7 days (SD: 1283.3). For the participants who remained free of dementia (*n* = 379), ‘time to dementia’ variable value was taken either as the time to death for those who died (*n* = 331, mean = 2863.0 days, SD: 1469.4), or time to a date beyond the last date of data collection for any participant for those who survived (*n* = 48; 6500 days).

### Main analyses

**Fig. 2:**
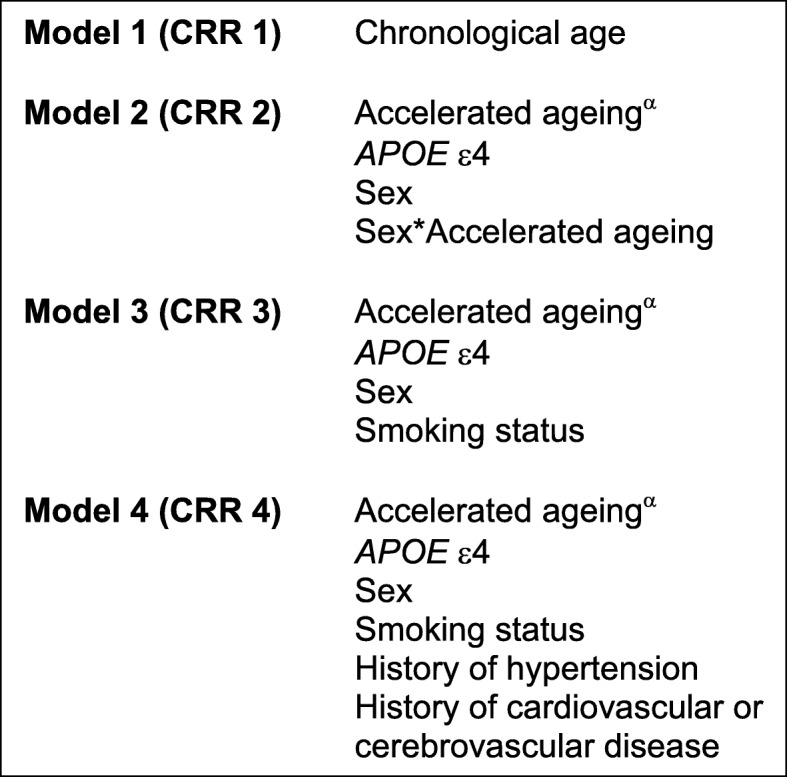
Competing Risk Regression Models. ^α^ Each model was repeated four times, each time substituting a different DNA methylation-based measure of accelerated ageing: EEAA, IEAA, AgeAccelPheno, AgeAccelGrim

The first competing risk model (CRR 1), included a single variable – chronological age (at baseline). In our study cohort (*n* = 488), chronological age at baseline did not demonstrate a statistically significant association with incident dementia (HR 1.00 [95% CI 1.00, 1.00], *p* = 0.61). Chronological age was not, therefore, included in subsequent competing risks models. The variables included in each model are shown in Fig. 2.

All subsequent models included a measure of accelerated ageing and each was completed four times – using EEAA, IEAA, AgeAccelPheno and AgeAccelGrim, in turn, as the measure of accelerated ageing (CRR models X_EEAA,_ X_IEAA,_ X_AgeAccelPheno_ and X_AgeAccelGrim_ respectively). The results for models 2–4 (for each age acceleration measure) are shown in Table 2. CRR 2 included four covariates: measure of accelerated ageing, sex, *APOE* ɛ4 carrier status and a sex by measure of accelerated ageing interaction term. In CRR 2_AgeAccelGrim_, where AgeAccelGrim was used as the measure of accelerated ageing, greater accelerated ageing was associated with lower risk of incident dementia (HR 0.89 (0.81, 0.97), *p* = 0.009). In the same model, carrying no *APOE* ɛ4 alleles was associated with a lower risk for incident dementia (HR 0.45 (0.30, 0.69), *p* < 0.001). A relationship between sex and incident dementia was not demonstrated (HR 0.84 (0.53, 1.34), *p* = 0.46). Similarly, the association between the sex by AgeAccelGrim interaction term and dementia did not reach statistical significance (HR 1.05 (0.94, 1.18), *p* = 0.38). When model 2 was repeated, using IEAA, EEAA and AgeAccelPheno (CRR 2_IEAA_, 2_EEAA_ and 2_AgeAccelPheno_ respectively), the association between accelerated ageing and dementia did not reach statistical significance. *APOE* ɛ4 negative carrier status associated with a lower risk for incident dementia in each of the three models. CRR 3 included the same covariates as model 2, with the addition of smoking status (ever smoker versus never smoker). Given its lack of statistical significance, the sex by age acceleration interaction term was dropped from subsequent models. In CRR 3_AgeAccelGrim_, the association between accelerated ageing (AgeAccelGrim) and dementia no longer reached statistical significance (HR 0.95 (0.89, 1.01), *p* = 0.09). Lifelong non-smoking (never smoking status) was associated with a higher risk of incident dementia (HR 1.69 (1.06, 2.71), *p* = 0.03). Negative *APOE* ɛ4 status continued to be associated with a lower risk of dementia (HR 0.44 (0.29, 0.67), *p* < 0.001). In CRR 3_IEAA_, 3_EEAA_ and 3_AgeAccelPheno_ there was no statistically significant relationship between accelerated ageing and dementia. Lifelong non-smoking status was associated with a higher risk of incident dementia in all three models; negative *APOE* ɛ4 carrier status was again associated with a lower risk of incident dementia in the three models. In CRR 4_AgeAccelGrim_ – which included history of hypertension, diabetes, cardiovascular or cerebrovascular disease as covariates – only *APOE* ɛ4 carrier status (HR 0.41 (0.27, 0.64), *p* < 0.001) and smoking status (HR 1.69 (1.05, 2.73), *p* = 0.03) reached statistical significance, with negative *APOE* ɛ4 carrier status reducing risk for incident dementia and never-smoking status increasing the risk for incident dementia. The same two variables reached statistical significance for the models including EEAA, IEAA and AgeAccelPheno as measures of accelerated ageing.

**Table 2:** Competing risk regression analyses results for EEAA, IEAA, AgeAccelPheno and AgeAccelGrim

	Hazard Ratios (95% Confidence Interval) for Probable Dementia											
	Results for EEAA			Results for IEAA			Results for AgeAccelPheno			Results for AgeAccelGrim		
	Model 2 (*n* = 383)	Model 3 (*n* = 382)	Model 4 (*n* = 371)	Model 2 (*n* = 383)	Model 3 (*n* = 382)	Model 4 (*n* = 371)	Model 2 (*n* = 383)	Model 3 (*n* = 382)	Model 4 (*n* = 371)	Model 2 (*n* = 383)	Model 3 (*n* = 382)	Model 4 (*n* = 371)
Measure of age acceleration	0.96 (0.92, 1.00)	0.98 (0.95, 1.01)	0.98 (0.95, 1.01)	0.97 (0.92, 1.02)	0.98 (0.95, 1.01)	0.98 (0.95, 1.01)	0.96 (0.93, 1.00)	0.99 (0.96, 1.02)	0.99 (0.96, 1.03)	0.89 (0.81, 0.97)	0.95 (0.89, 1.01)	0.95 (0.89, 1.01)
Sex (female)	0.92 (0.58, 1.44)	0.87 (0.55, 1.40)	0.87 (0.53, 1.41)	1.02 (0.66, 1.59)	0.93 (0.60, 1.44)	0.94 (0.56, 1.47)	1.06 (0.68, 1.63)	0.97 (0.63, 1.49)	0.97 (0.62, 1.52)	0.84 (0.53, 1.34)	0.83 (0.51, 1.36)	0.84 (0.51, 1.38)
*APOE* ɛ4 (non-carrier)	0.43 (0.28, 0.65)	0.42 (0.28, 0.64)	0.40 (0.26, 0.60)	0.43 (0.28, 0.67)	0.43 (0.28, 0.65)	0.40 (0.26, 0.61)	0.43 (0.28, 0.67)	0.43 (0.28, 0.65)	0.40 (0.26, 0.61)	0.45 (0.30, 0.69)	0.44 (0.29, 0.67)	0.41 (0.27, 0.64)
Age acceleration by Sex interaction term	1.03 (0.97, 1.09)	–	–	1.01 (0.95, 1.08)	–	–	1.04 (0.98, 1.10)	–	–	1.05 (0.94, 1.18)	–	–
Smoker (never)	–	2.00 (1.30, 3.07)	1.97 (1.27, 3.06)	–	2.01 (1.31, 3.09)	1.98 (1.28, 3.07)	–	2.02 (1.30, 3.14)	2.00 (1.27, 3.17)	–	1.69 (1.06, 2.71)	1.69 (1.05, 2.73)
History of hypertension	–	–	0.73 (0.47, 1.15)	–	–	0.75 (0.48, 1.17)	–	–	0.74 (0.47, 1.16)	–	–	0.71 (0.45, 1.12)
History of diabetes	–	–	1.24 (0.44, 3.44)	–	–	1.23 (0.45, 3.40)	–	–	1.22 (0.44, 3.42)	–	–	1.20 (0.44, 3.30)
History of cardiovascular or cerebrovascular disease	–	–	0.75 (0.44, 1.28)	–	–	0.74 (0.44, 1.27)	–	–	0.74 (0.44, 1.27)	–	–	0.75 (0.44, 1.27)

Each model was repeated as a logistic regression model, with probable dementia as the outcome. In these analyses – where death was not considered – the association between AgeAccelGrim and dementia reached statistical significance in models 2 and 3 (*p* < 0.05) and approached significance in model 4 (*p* = 0.06). In each case, greater age acceleration reduced the risk for subsequent dementia. The association between AgeAccelPheno, EEAA, IEAA and dementia was not statistically significant in any model. Being a non-carrier for the *APOE* ε4 allele was associated with a reduced risk for dementia in every model (*p* < 0.001). Being a never-smoker increased the risk for dementia in every model where it was included (*p* < 0.05). The complete results for the logistic regression are available in Additional file 2: Table S1.

### Components of AgeAccelGrim

Given that the association observed between AgeAccelGrim and dementia was the only significant finding regarding the age acceleration measures when examined using competing risk regression analyses (CRR 2_AgeAccelGrim_), and that the direction of association was opposite to what we might have expected, we wished to investigate it further. Based on the change in statistical significance observed following the introduction of a smoking variable, we hypothesized that the association seen had been related to the smoking component of the age acceleration measure. By separating out the individual components of AgeAccelGrim, we were able to look at the association between the DNA methylation-based surrogate biomarker for smoking pack years and dementia on its own to see if any association we were seeing for the measure overall was mirrored in what was observed for this component. The components on which the AgeAccelGrim measure was based were therefore considered in turn. Model 2 was repeated eight times, each time substituting a component of the measure for AgeAccelGrim. The components included DNA methylation-based surrogate markers for smoking pack years (DNAm PACKYRS) and seven plasma proteins – beta-2 microglobulin (DNAm B2M), cystatin-C (DNAm Cystatin C), growth differentiation factor 15 (DNAm GDF-15), plasma activator-inhibitor 1 (DNAm PAI-1), Leptin (DNAm Leptin), adrenomedullin (DNAm ADM), and tissue inhibitor metalloproteinase 1 (DNAm TIMP-1). Each was entered into a competing risk regression model along with *APOE* ɛ4 carrier status and sex. An interaction term was not included given that it was not previously found to be statistically significant. Only the association between the DNAm PACKYRS component and dementia reached statistical significance (HR: 0.97 [0.95, 0.99], *p* = 0.007). The complete results for these component analyses are provided in Additional file 3: Table S2.

These analyses were again repeated as logistic regression analyses. The association between DNAm PACKYRS and dementia once again reached statistical significance (*p* < 0.05). The complete results for these analyses are provided in Additional file 4: Table S3.

### Cumulative incidence graphs

**Fig. 3:**
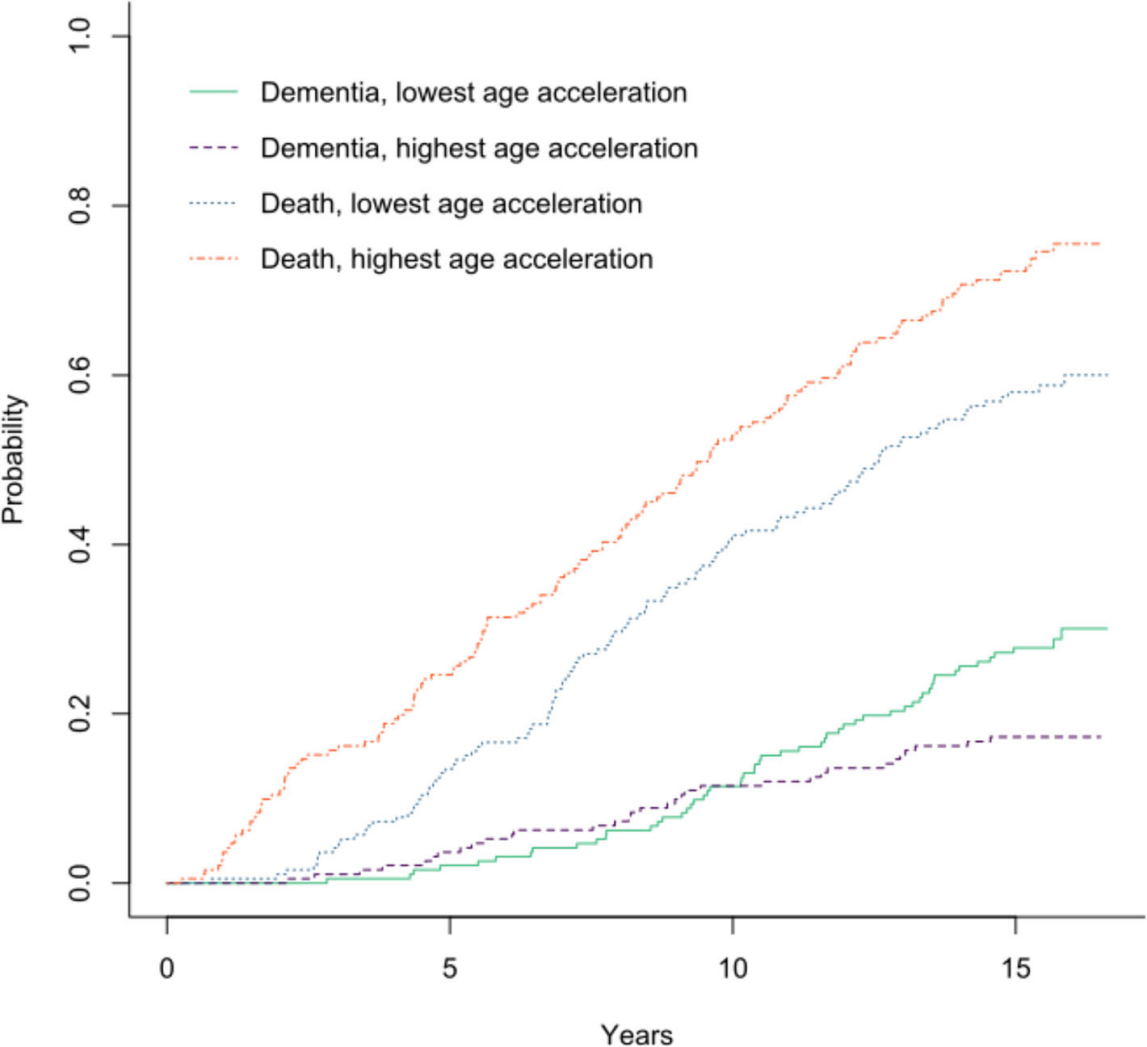
Cumulative incidence plots for AgeAccelGrim: death and dementia. *Note.* The two groups for each event (death and dementia) were formed from the half of participants with higher age acceleration levels who experienced that event and the half with lower age acceleration levels that experienced that same event. A steeper rising line indicates that individuals in this group were at greatest risk for the event, e.g., in general, individuals were more at risk for death than dementia

Figure 3 shows cumulative incidence plots for the two competing events: dementia and death. The general direction of the cumulative incidence plot does seem to support the direction of the association observed between AgeAccelGrim and dementia in CRR 1_AgeAccelGrim_, with increased risk for dementia for those with lower levels of age acceleration. Based on the figure, it would appear that the reversal and divergence of the association between age acceleration and dementia begins 10 years after baseline, when subjects are aged approximately 89 years. In contrast with the unclear pattern demonstrated for dementia, the cumulative incidence plot indicated a greater risk for death for those with highest levels of accelerated ageing (calculated using AgeAccelGrim in our study) compared with those with lowest levels of age acceleration. This result reinforced the patterns of association shown in previous studies, where higher age acceleration was associated with a greater risk of mortality [7].

### Correlation between measures of age acceleration

In this study cohort, positive correlations were shown between each of the four measures of epigenetic age acceleration (0.259 ≤ *r* ≤ 0.439) (Additional file 5: Table S4). The strongest correlation was seen between EEAA and AgeAccelGrim (*r* = 0.439), but this was only marginally greater than the correlations observed between EEAA and AgeAccelPheno (*r* = 0.424), AgeAccelPheno and AgeAccelGrim (*r* = 0.416) and AgeAccelPheno and IEAA (*r* = 0.403). The weakest correlation was seen between IEAA and AgeAccelGrim (*r* = 0.259); this may reflect the fact that IEAA is based on Horvath’s original measure which was developed using multiple tissue types, while AgeAccelGrim was developed using blood methylation data alone [12].

## Discussion

This study aimed to determine whether DNA methylation-based measures of accelerated ageing were associated with risk for dementia in the oldest-old. The results did not demonstrate any consistent relationship between DNA-based measures of accelerated ageing and incident dementia. The initial findings suggested that increased AgeAccelGrim may be associated with decreased risk for incident dementia in those aged over 79 years. However, on subsequent testing the results indicated that smoking might explain this association, and more specifically, that it was likely to be the collinear relationship between GrimAge and smoking that had given rise to this finding.

### Comparison with previous findings

Whereas a number of studies have suggested an increased risk for dementia associated with DNA methylation patterns at specific loci or with increased DNA methylation age, we are not aware of any studies that specifically examined the relationship between methylation-based measures of accelerated ageing and dementia [28–31]. For this reason, it is not possible for us to directly compare our findings. We can however consider previous notable findings relating DNA methylation and dementia.

Investigating how a change in the expression of DNA alters the risk of developing the dementia is of clear value in both furthering our understanding of the pathogenesis of the disease and in guiding the development of effective treatments. To this end, several studies have considered how specific changes in DNA methylation affect one’s risk of developing dementia, with notable epigenetic changes being observed between subjects with dementia and controls [30]. Recent studies have indicated that DNA methylation may indeed contribute to the pathogenesis of dementia. For example, the *APOE* gene (variants of which are recognised to be important in dementia risk) has been shown to be differently methylated in Alzheimer’s disease (AD) [32]. Specifically, reduced methylation levels at a well-defined CpG island within the fourth exon of the *APOE* gene in brain tissue were observed in AD subjects when compared with controls; these differences in methylation levels were observed in both the hippocampus and frontal lobe regions of the brain, where AD pathophysiological changes were abundant [32]. Furthermore, DNA methylation levels were increased in the presence of an *APOE* ɛ4 allele in controls, but not in AD subjects [32]. Studies have also reported changes in DNA methylation, in relation to AD, at several other genes [30]. In 2014, De Jager et al. and Lunnon et al. published the results of two large-scale epigenome-wide association studies (EWAS) in Alzheimer’s disease [33–35]. Differences in methylation were reported at a number of loci, including four that were independently identified in both studies: *ANK1, RPL13, C10orf54-CDH23* and *RHBDF2* [33–35]. A 2016 systematic review by Wen et al. described studies reporting higher methylation levels of several genes (observed in peripheral blood cells or brain tissue of AD patients) including *OPRK1*, *BDNF*, *UQCRC1*, *HTERT*, *TREM2*, *TBX2AR*, *SORBS3*, *SPTBN4*, and *CREB* promotors and the synaptophysin gene [30]. Lower levels of methylation of a number of other genes were reported in the blood or brain tissues of AD subjects, including *PIN1*, *FAAH*, *ALOX5*, *DR4*, *TNFA*, *COX-2*, *NF-kβ*, *CRTC1* and *S100A2* [30]. Other studies have also suggested differences in global DNA methylation – i.e. the overall level of methylcytosine within the genome – between AD subjects and controls [30]. While the results described are not consistent across all studies, the evidence would seem to support the hypothesis that DNA methylation plays an important role in dementia.

Findings relating to DNAm age and dementia are of particular relevance here given the direct relationship between DNA methylation-based age and measures of accelerated ageing. Levine at al. (2018) tested for an association between pathologically determined AD and DNAm PhenoAge in the dorsolateral prefrontal cortex [11]. They found that when comparing same-age individuals, the dorsolateral prefrontal cortex appeared more than one year older in those with AD [11]. Furthermore, DNAm PhenoAge was associated with typical neuropathological signs of AD including neurofibrillary tangles, amyloid load and neuritic plaques [11]. A previous Swedish longitudinal study examined the association between DNAm age (calculated using Hovarth’s epigenetic clock) and dementia and the authors reported that increased DNAm age was a statistically significant predictor for dementia (*β* = 0.16, *p* = 0.019) [28]. This was however a small study, with *n* = 11 dementia cases, and the logistic regression analyses were adjusted for gender only [28]. DNAm age was calculated at a time when *n* = 6 of these cases were already diagnosed, and *n* = 5 were diagnosed in the following four years [28]. We must therefore consider whether this study describes an association between advanced DNAm age in existing dementia, as opposed to increased DNAm age predicting dementia.

Based on these previous findings, one might have expected that the present study would have identified a similar association between accelerated ageing and dementia. There may be a number of reasons why this was not the case, and these are discussed within the context of the mechanisms and limitations of the study below.

### Mechanisms

Whereas chronological age is widely recognised to be associated with dementia, this was not the case in our study cohort. It is probable that the absence of such an association in this study can be attributed to the use of a narrow-age cohort. In studies of methylation age and dementia using participants of a wider age-range, chronological age would likely be an important covariate for inclusion. As has been shown previously in this study cohort [22, 27], the presence of at least one *APOE* ɛ4 allele was related to an increased risk of incident dementia. As such, it remains an important covariate for inclusion in studies of dementia in those aged over 80 years.

The results of the analyses included in this study did not find any consistent relationship between DNA-based measures of accelerated ageing and incident dementia. Only one model yielded a result for accelerated ageing that reached statistical significance at conventional levels. Indeed, this result contradicted the hypothesised results, with increased accelerated ageing (AgeAccelGrim) being associated with a reduced risk for incident dementia. This finding would contradict those previous studies that have shown an association between increased methylation age and greater risk for developing age-related health outcomes [6, 7]. Given the unexpected direction of this association, one must consider the robustness of this finding. Given that the magnitude of the association was relatively small and was not observed in any subsequent model it may be that this was a chance finding that does not demonstrate a true association. In this study, the introduction of a smoking variable meant that the association between AgeAccelGrim and dementia no longer reached statistical significance. Such a finding might be expected given that GrimAge is built, in part, on smoking related data and the two are extremely collinear, correlating at approximately 0.9. We note that the direction of association between smoking and dementia in this study is the same as that for AgeAccelGrim and dementia. We therefore suggest that an association between smoking and dementia seems to explain the observed relationship between AgeAccelGrim and dementia. Our finding that the DNA methylation-based marker for smoking pack years was the only component of AgeAccelGrim associated with dementia in this cohort provided further evidence for this explanation.

In these analyses, a lifelong history of non-smoking was associated with an increased risk for dementia. While the direction of this association may defy the expected and contradict previous studies, it is in line with a general pattern observed in this cohort of individuals aged over 79 years [22]. A previous study of the LBC1921 has also demonstrated an increased risk for dementia after age 79 years with greater lifetime physical activity, and a decreased risk for dementia for those with a history of hypertension at age 79 years [22]. Similarly, other factors that have previously been shown to increase risk for dementia in studies of earlier old age have been found to have no effect on risk in this cohort of participants aged over 79 years [22, 27]. In a previous study of physical fitness and dementia in the LBC1921, a positive history of ever-smoking was observed to decrease risk for dementia, but in that study the association did not reach statistical significance [27]. It would therefore appear that the statistical significance of the association between ever-smoking and dementia within our cohort is dependent on the covariates included in the analyses. It is possible that the direction of the association between age acceleration and dementia observed in these analyses simply reflects of the direction of the association between smoking and dementia in this this cohort, but we acknowledge that the inconsistency in statistical significance means that we must treat the association observed in this study with caution. We must also consider whether survival to age 79 years or recruitment at age 79 years have influenced our smoking-related findings. It is possible that those individuals who were most likely to have experienced greater risk for dementia as a result of previous or current smoking had died earlier to age 79 years, leaving only those who would remain unaffected or in some way ‘resistant’ to the negative effects of smoking. Similarly, we must consider the possibility that those who would have been more likely to develop dementia a result of their smoking history had done so prior to recruitment age and would not therefore have been eligible to enrol in the LBC1921 study. Given that susceptibility for lung disease is variable between persons [36], one might suggest that there is a similar variability in susceptibility for dementia and those who remained dementia free at age 79 would be those with a reduced susceptibility, giving rise to an apparent reduction in risk for smokers.

### Implications

Without any consistent results it is difficult to draw any comparisons between the age acceleration measures considered in this study, and how useful each might be in establishing risk for incident dementia. Furthermore, the lack of positive findings regarding dementia in the present study limits the clinical implications specific to dementia. There is a clear requirement for further study in this field; a full appreciation of the role of DNA methylation and DNA methylation-based measures of accelerated ageing in dementia could be of considerable value in furthering our understanding of the risks for dementia and identifying potential targets for risk reduction. Our null finding might initially suggest that future similar studies were not required. Given some of the limitations of our study cohort however, we cannot assume that our study answers this research question conclusively, particularly given that this has not been investigated previously within the literature. Our suggestion for larger studies would be to overcome the potential limitations of our study that may have given rise to the null finding. A stand-alone study can rarely be taken as conclusive evidence and additional studies would therefore either add strength to, or refute, our null finding.

### Strengths and limitations

The study cohort used in these analyses has a number of strengths. The LBC1921 is a narrow-age cohort of persons aged 79 years of age at baseline; this means that the study does not suffer from the major confounding effect of chronological age. As such, the cohort is suited to the study of dementia in the oldest-old. Given the homogeneous nature of the cohort participants, confounding errors resulting from age, ethnic, cultural and geographical variability would be unlikely. Participants have taken part in a detailed longitudinal follow-up procedure, and death ascertainment for the cohort is complete. Previously published assessments of validation have shown the dementia ascertainment methods used in this study to be effective and incidence rates to be comparable with expected rates for the cohort [22]. We cannot however exclude the possibility of missed or misclassified cases of dementia in our cohort. In particular, a limitation of our study is the potential that we missed cases of preclinical or prodromal dementia in participants who died prior to developing clinical dementia. In addition to this, it is possible that cases of preclinical or prodromal dementia present at the time we concluded our ascertainment would have gone on to develop dementia after that date.

The indication of a possible reverse association (to that which was expected) for AgeAccelGrim, combined with the surprising association with never smoking (in addition to those unusual associations observed in previous manuscripts), could suggest a cohort effect; it is possible that something specific about this study sample – such as a survivor bias, or something else about the nature of recruitment – may have influenced the results.

A *p* value of 0.05 was used to determine significance for all models. We did not therefore specifically compensate for potential erroneous inferences arising from multiple testing.

Whereas the LBC1921 is a detailed cohort, it is however limited in size. Studies of DNA methylation-based measures of accelerated ageing and dementia within larger cohorts are required to provide further evidence in this field. As noted above, there were insufficient numbers of eligible study subjects to further investigate whether DNA methylation age mediates the risk of smoking. This study cohort did not have a sufficient number of confirmed cases of dementia of the Alzheimer’s type to allow for a specific analysis of this outcome. Again, larger studies with subjects who had confirmed dementia aetiology would allow for such analyses.

Another strength of this study was the inclusion of death as a competing risk. We hypothesised that death could affect the results, given the recognised association between age acceleration and mortality. When we repeat model 2_AgeAccelGrim_ as a simple Cox regression analysis (without death as a competing risk), the effect size for AgeAccelGrim was reduced and did not reach statistical significance (HR 0.99 (0.93, 1.06)).

In order to complete our study using a competing risks type analyses, it was necessary to have ‘time to event’ variables, including a ‘time to dementia’ for those who developed the condition during the follow-up period. We have described within the methods the manner by which such estimates were determined for these analyses. We must however recognise the uncertainty that exists regarding the accuracy of this estimate. The nature of dementia and the often gradual onset mean that it is difficult to pin-point an exact date of onset. Furthermore, the variability in how each individual perceives their own symptoms and the differing stages at which one may present for cognitive assessment mean that when diagnoses are ascertained from records, time of onset may be even harder to determine. Depending on the source of data available for dementia ascertainment, the date of diagnosis was not always listed, making it even more difficult to calculate a time to dementia estimate. In this study, the methods for calculating the time to dementia aimed to provide the most accurate estimate that was possible using the available information, but the potential for inaccurate estimates to have affected the results is acknowledged.

Finally, while we must consider the potential for inaccuracy in self-reported smoking data. This is however less relevant in this study given the accuracy of the DNA methylation based marker for smoking.

## Conclusion

In conclusion, the present study did not demonstrate any consistent association between DNA methylation-based measures of accelerated ageing and dementia in subjects aged over 79 years. Further, larger studies – including analyses of separate dementia subtypes – are required to further investigate the potential association between DNA methylation-based measures of accelerated ageing and dementia.

## Supplementary information

**Additional file 1: Figure S1.** ICD-9 and ICD-10 codes relevant to dementia ascertainment.

**Additional file 2: Table S1.** Logistic Regression Analyses Results for EEAA, IEAA, AgeAccelPheno and AgeAccelGrim.

**Additional file 3: Table S2.** Competing risk regression models for components of AgeAccelGrim.

**Additional file 4: Table S3.** Logistic regression models for components of AgeAccelGrim.

**Additional file 5: Table S4.** Pearson correlations for epigenetic age acceleration measures in LBC1921.

## Data Availability

The data utilised and described within this study are available on request from the Lothian Birth Cohort Study, Centre for Cognitive Ageing and Cognitive Epidemiology, University of Edinburgh. The data are not publically available due to them containing information that might compromise participant confidentiality and consent.

## Abbreviations

AgeAccelGrim: Age acceleration calculated from GrimAge
AgeAccelPheno: Age acceleration calculated from PhenoAge
*APOE* ɛ4: Apolipoprotein E ɛ4
CHI: Community Health Index
CI: Confidence Interval
CRR: Competing risk regression model
DNA: Deoxyribonucleic Acid
DNAm: DNA methylation
EEAA: Extrinsic Epigenetic Age Acceleration
GrimAge: DNA methylation age, calculated using the GrimAge procedure
HR: Hazard Ratio
IEAA: Intrinsic Epigenetic Age Acceleration
LBC1921: Lothian Birth Cohort 1921
MHT: Moray House Test
MMSE: Mini Mental State Examination
PhenoAge: DNA methylation age, calculated using the PhenoAge procedure
SD: Standard Deviation
SMS1932: Scottish Mental Survey 1932
