# Supplementary material for: DNA methylation-based measures of accelerated biological ageing and the risk of dementia in the oldest-old: a study of the Lothian Birth Cohort 1921
Source: BMC Psychiatry. 2020 Feb 28;20:91. doi: 10.1186/s12888-020-2469-9 (PMC7048023; doi:10.1186/s12888-020-2469-9)
Supplement: Supplementary file 1 — Additional file 1: Figure S1. ICD-9 and ICD-10 codes relevant to dementia ascertainment. [file 12888_2020_2469_MOESM1_ESM.docx]

**Additional file 1: Figure S1.** ICD-9 and ICD-10 codes relevant to dementia ascertainment

| **ICD-9 Codes**  **046.1** Creutzfeldt-Jakob disease  **290.0** Senile dementia, simple type  **290.1** Presenile dementia  **290.2** Senile dementia, depressed or paranoid type  **290.3** Senile dementia with acute confusional state  **290.4** Arteriosclerotic dementia  **290.8** Other senile and presenile organic psychotic conditions  **290.9** Unspecified senile and presenile organic psychotic conditions  **291.2** Other alcoholic dementia  **292.82** Drug induced persisting dementia  **294.0** Korsakov’s psychosis, alcoholic  **294.1** Dementia in conditions classified elsewhere  **294.8** Other organic psychotic conditions: Other  **294.9** Other organic psychotic conditions: Unspecified  **331.0** Alzheimer’s disease  **331.1** Pick’s disease  **331.2** Senile degeneration of the brain  **331.82** Dementia: Lewy body  **333.4** Huntington’s chorea  **797** Senility without mention of psychosis | **ICD-10 Codes**  **F00** Dementia in Alzheimer’s disease  **F01** Vascular dementia  **F01.1** Multi-infarct dementia  **F02** Dementia in diseases classified elsewhere  **F02.0** Dementia in Pick’s disease  **F02.1** Dementia in Creutzfeldt-Jakob disease  **F02.2** Dementia in Huntington’s disease  **F02.3** Dementia in Parkinson’s disease  **F02.4** Dementia in HIV  **F03**  Unspecified dementia  **F05.1** Delirium superimposed on dementia  **F06.7** Mild cognitive disorder  **G30** Alzheimer disease  **G30.0** Alzheimer disease with early onset  **G30.1** Alzheimer disease with late onset  **G30.8** Other Alzheimer disease  **G30.9** Alzheimer disease, unspecified  **G31.0** Circumscribed brain atrophy: Frontotemporal dementia, Pick disease, progressive isolated aphasia  **G31.1** Senile degeneration of the brain not classified elsewhere  **G31.8** Other specified degenerative diseases of nervous system: Grey-matter degeneration, Lewy body(dies)(dementia)(disease), subacute necrotising encephalopathy |
| --- | --- |

(ICD9Data.com; World Health Organisation, 2016)
