# Supplementary material for: DNA methylation-based measures of accelerated biological ageing and the risk of dementia in the oldest-old: a study of the Lothian Birth Cohort 1921
Source: BMC Psychiatry. 2020 Feb 28;20:91. doi: 10.1186/s12888-020-2469-9 (PMC7048023; doi:10.1186/s12888-020-2469-9)
Supplement: Supplementary file 2 — Additional file 2: Table S1. Logistic Regression Analyses Results for EEAA, IEAA, AgeAccelPheno and AgeAccelGrim. [file 12888_2020_2469_MOESM2_ESM.docx]

**Additional file 2: Table S1:** Logistic Regression Analyses Results for EEAA, IEAA, AgeAccelPheno and AgeAccelGrim

|  | **Odds Ratios (95% Confidence Interval)**  **for Probable Dementia** | | | | | | | | | | | |
| --- | --- | --- | --- | --- | --- | --- | --- | --- | --- | --- | --- | --- |
|  | Results for EEAA | | | Results for IEAA | | | Results for AgeAccelPheno | | | Results for AgeAccelGrim | | |
|  | Model 2  (*n*=383) | Model 3  (*n*=382) | Model 4  (*n*=371) | Model 2  (*n*=383) | Model 3  (*n*=382) | Model 4  (*n*=371) | Model 2  (*n*=383) | Model 3  (*n*=382) | Model 4  (*n*=371) | Model 2  (*n*=383) | Model 3  (*n*=382) | Model 4  (*n*=371) |
| Measure of age acceleration | -0.05  (-0.11, 0.00) | -0.03  (-0.07, 0.00) | -0.03  (-0.07, 0.01) | -0.04  (-0.11, 0.03) | -0.03  (-0.07, 0.01) | -0.02  (-0.07, 0.02) | -0.05  (-0.10, 0.00) | -0.02  (-0.06, 0.02) | -0.02  (-0.05, 0.02) | -0.14  (-0.27, -0.03) | -0.08  (-0.15, -0.01) | -0.07  (-0.14, 0.00) |
| Sex  (female) | -0.16  (-0.70, 0.40) | -0.21  (-0.76, 0.34) | -0.20  (-0.78, 0.37) | -0.03  (-0.54, 0.50) | -0.14  (-0.66, 0.40) | -0.11  (-0.66, 0.44) | -0.00  (-0.51, 0.51) | -0.09  (-0.61, 0.43) | -0.07  (-0.61, 0.48) | -0.27  (-0.82, 0.27) | -0.27  (-0.83, 0.29) | -0.24  (-0.81, 0.34) |
| *APOE* ɛ4  (non-carrier) | -0.99  (-1.49, -0.48) | -0.99  (-1.51, -0.47) | -1.09  (-1.63, -0.55) | -0.95  (-1.46, -0.44) | -0.96  (-1.48, -0.45) | -1.06  (-1.60, -0.53) | -0.94  (-1.45, -0.43) | -0.95  (-1.47, -0.43) | -1.0  (-1.59, -0.52) | -0.91  (-1.42, -0.39) | -0.93  (-1.45, -0.41) | -1.03  (-1.57, -0.49) |
| Age acceleration by  Sex interaction term | 0.02  (-0.05, 0.10) | - | - | 0.01  (-0.07, 0.10) | - | - | 0.04  (-0.03, 0.12) | - | - | 0.06  (-0.08, 0.20) | - | - |
| Smoker (never) | - | 0.79  (0.29, 1.30) | 0.79  (0.27, 1.32) | - | 0.79  (0.29, 1.30) | 0.79  (0.27, 1.32) | - | 0.80  (0.30, 1.31) | 0.80  (0.28, 1.33) | - | 0.57  (0.02, 1.12) | 0.58  (0.02, 1.16) |
| History of hypertension | - | - | -0.43  (-0.99, 0.10) | - | - | -0.42  (-0.97, 0.12) | - | - | -0.42  (-0.98, 0.11) | - | - | -0.47  (-1.02, 0.07) |
| History of diabetes | - | - | 0.31  (-1.02, 1.44) | - | - | 0.31  (-1.02, 1.43) | - | - | 0.31  (-1.02, 1.44) | - | - | 0.27  (-1.07, 1.40) |
| History of cardiovascular or cerebrovascular disease | - | - | -0.33  (-0.95, 0.26) | - | - | -0.34  (-0.96, 0.25) | - | - | -0.33  (-0.95, 0.26) | - | - | -0.33  (-0.95, 0.26) |
