# Supplementary material for: DNA methylation-based measures of accelerated biological ageing and the risk of dementia in the oldest-old: a study of the Lothian Birth Cohort 1921
Source: BMC Psychiatry. 2020 Feb 28;20:91. doi: 10.1186/s12888-020-2469-9 (PMC7048023; doi:10.1186/s12888-020-2469-9)
Supplement: Supplementary file 3 — Additional file 3: Table S2. Competing risk regression models for components of AgeAccelGrim. [file 12888_2020_2469_MOESM3_ESM.docx]

**Additional file 3: Table S2.** Competing risk regression models for components of AgeAccelGrim

|  | **Component of AgeAccelGrim** | | | | | | | |
| --- | --- | --- | --- | --- | --- | --- | --- | --- |
|  | **DNAm ADM** | **DNAm B2M** | **DNAm CystatinC** | **DNAm GDF15** | **DNAm Leptin** | **DNAm PAI1** | **DNAm TIMP1** | **DNAm PACKYRS** |
| Component of AgeAccelGrim | 0.99  (0.98, 1.00) | 1.00  (1.00, 1.00) | 1.00  (1.00, 1.00) | 1.00  (1.00, 1.00) | 1.00  (1.00, 1.00) | 1.00  (1.00, 1.00) | 1.00  (1.00, 1.00) | 0.97  (0.95, 0.99) |
| Sex  (female) | 1.33  (0.82, 2.16) | 1.10  (0.72, 1.70) | 1.07  (0.68, 1.69) | 1.07  (0.69, 1.66) | 0.86  (0.39, 1.89) | 1.13  (0.72, 1.77) | 1.04  (0.67, 1.61) | 0.94  (0.60, 1.47) |
| *APOE* ɛ4  (non-carrier) | 0.43  (0.28, 0.65) | 0.43  (0.28, 0.65) | 0.43  (0.28, 0.66) | 0.44  (0.29, 0.67) | 0.43  (0.28, 0.66) | 0.43  (0.28, 0.65) | 0.44  (0.29, 0.67) | 0.46  (0.30, 0.70) |
