# Supplementary material for: DNA methylation-based measures of accelerated biological ageing and the risk of dementia in the oldest-old: a study of the Lothian Birth Cohort 1921
Source: BMC Psychiatry. 2020 Feb 28;20:91. doi: 10.1186/s12888-020-2469-9 (PMC7048023; doi:10.1186/s12888-020-2469-9)
Supplement: Supplementary file 4 — Additional file 4: Table S3. Logistic regression models for components of AgeAccelGrim. [file 12888_2020_2469_MOESM4_ESM.docx]

**Additional file 4: Table S3.** Logistic regression models for components of AgeAccelGrim

|  | **Component of AgeAccelGrim** | | | | | | | |
| --- | --- | --- | --- | --- | --- | --- | --- | --- |
|  | **DNAm ADM** | **DNAm B2M** | **DNAm CystatinC** | **DNAm GDF15** | **DNAm Leptin** | **DNAm PAI1** | **DNAm TIMP1** | **DNAm PACKYRS** |
| Component of AgeAccelGrim | -0.01  (-0.02, 0.00) | -1.39  (-3.85, 9.33) | -6.94  (-1.80, 3.44) | -0.00  (-0.00, 0.00) | 3.88  (-6.45, 0.00) | 1.39  (-1.94, 2.09) | -0.00  (-0.00, 0.00) | -0.04  (-0.06, -0.01) |
| Sex  (female) | 0.29  (-0.27, 0.86) | 7.11  (-4.26, 5.79) | 3.54  (-4.67, 5.47) | 0.03  (-0.47, 0.54) | -1.95  (-1.07, 0.68) | 9.58  (-0.42, 0.62) | -0.03  (-0.54, 0.49) | -0.12  (-0.64, 0.41) |
| *APOE* ɛ4  (non-carrier) | -0.97  (-1.48, -0.46) | -9.69  (-1.47, -4.63) | -9.70  (-1.48, -4.64) | -0.95  (-1.45, -0.44) | -9.58  (-1.46, -0.45) | -9.71  (-1.48, -0.47) | -0.96  (-1.46, -0.45) | -0.89  (-1.40, -0.38) |
