# Supplementary material for: DNA methylation-based measures of accelerated biological ageing and the risk of dementia in the oldest-old: a study of the Lothian Birth Cohort 1921
Source: BMC Psychiatry. 2020 Feb 28;20:91. doi: 10.1186/s12888-020-2469-9 (PMC7048023; doi:10.1186/s12888-020-2469-9)
Supplement: Supplementary file 5 — Additional file 5: Table S4. Pearson correlations for epigenetic age acceleration measures in LBC1921. [file 12888_2020_2469_MOESM5_ESM.docx]

**Additional file 5: Table S4.** Pearson correlations for epigenetic age acceleration measures in LBC1921

|  | **IEAA** | **EEAA** | **AgeAccelGrim** | **AgeAccelPheno** |
| --- | --- | --- | --- | --- |
| **IEAA** | 1 | 0.394* | 0.259* | 0.403* |
| **EEAA** | 0.394* | 1 | 0.439* | 0.424* |
| **AgeAccelGrim** | 0.259* | 0.439* | 1 | 0.416* |
| **AgeAccelPheno** | 0.403* | 0.424* | 0.416* | 1 |

*Note. n=383. *Correlation is significant at the 0.01 level (2-tailed)*
